# Novel insights into how the mean and heterogeneity of abiotic conditions together shape forb species richness patterns in the Allegheny plateau ecoregion

**DOI:** 10.1002/ece3.5508

**Published:** 2019-09-30

**Authors:** Samantha A. Catella, Sarah R. Eysenbach, Karen C. Abbott

**Affiliations:** ^1^ Abbott Lab Case Western Reserve University Cleveland Ohio; ^2^ Cleveland Metroparks Cleveland Ohio

**Keywords:** abiotic heterogeneity, available energy hypothesis, environmental gradient, herbaceous layer, heterogeneity–diversity relationship hypothesis, plant community structure

## Abstract

**Abstract:**

While plant community theory tends to emphasize the importance of abiotic heterogeneity along niche axes, much empirical work seeks to characterize the influence of the absolute magnitude of key abiotic variables on diversity. Both magnitude (as reflected, e.g., by a mean) and heterogeneity (variance) in abiotic conditions likely contribute to biodiversity patterns in plant communities, but given the large number of putative abiotic drivers and the fact that each may vary at different spatiotemporal scales, the challenge of linking observed biotic patterns with the underlying environment remains acute. Using monitoring data from a natural resource agency, we compared how well statistical models of the mean, heterogeneity, and both the mean and heterogeneity combined of 17 abiotic factor variables explained patterns of forb species richness in Northeast Ohio, USA. We performed our analyses at two spatial scales, repeated in spring and summer across four forest types. Although all models explained a great deal of the variance in species richness, models including both the mean and heterogeneity of different abiotic factors together outperformed models including either the mean or the heterogeneity of abiotic factors alone. Variability in forb species richness was mostly due to changes in mean calcium levels regardless of forest type. After accounting for forest type, we were able to attribute variation in forb species richness to changes in the heterogeneity of different abiotic factors as well. Our results suggest that multiple mechanisms act simultaneously according to different aspects of the abiotic environment to structure forb communities, and this underscores the importance of considering both the magnitude of and heterogeneity in multiple abiotic factors when looking for links between the abiotic environment and plant community patterns. Finally, we identify novel patterns across spatial scales, forest types, and seasons that can guide future research in this vein.

**OPEN RESEARCH BADGES:**

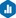

This article has earned an Open Data Badge for making publicly available the digitally‐shareable data necessary to reproduce the reported results. The data is available at https://doi.org/10.5061/dryad.kp3cb17.

## Graphical abstract

Herbaceous communities are an important component of temperate deciduous hardwood forests. However, it remains unclear how patterns of species richness change according to mean abiotic conditions compared to the heterogeneity in abiotic conditions. Using the mean and heterogeneity of 17 different abiotic factors measured in forests in Northeast Ohio, we used statistical models to ask which abiotic factor measurements explained the most variation in species richness within and across different forest community types at two spatial scales, and in spring compared to summer. We found that although mean calcium conditions shaped species richness across forests, heterogeneity in various abiotic factor conditions shaped species richness within forests.

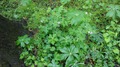

## 1 INTRODUCTION

A longstanding goal in ecology is to understand how species' responses to the environment will scale up to community‐level patterns (Braun, 1950; Cornell & Lawton, 1992; HilleRisLambers, Adler, Harpole, Levine, & Mayfield, 2012; Raup, 1942; Tilman, 1982). However, it is often difficult to incorporate realistic abiotic complexity into research questions because organisms can respond to multiple abiotic factors in different ways. For example, species in a community might show classic niche differentiation with respect to one abiotic variable (McKane et al., 2002), while simultaneously sharing a single niche optimum for some other variable (Vergnon, Ooi, & Freckleton, 2017). Individual abiotic factors vary through time and have unique spatial characteristics, differing in their range of variation and scale of spatial autocorrelation. Given all this complexity, it is easy to see why the role of abiotic drivers is often simplified, especially in research emphasizing other aspects of plant community structure. Establishing how plant communities are impacted by multiple abiotic factors that vary at different spatial and temporal scales is nevertheless a crucial step toward a more complete picture of spatial plant ecology.

Recent simulation work has shown promise in using spatial statistics to distinguish between plant community patterns that arise from different mechanisms (Brown, Illian, & Burslem, 2016). However, as the authors point out, ignoring the complexity of the abiotic environment will likely limit the application of this approach to real systems, because the signature that results from a given biotic mechanism may depend on the spatial structure of the abiotic environment. In other words, regardless of whether our central interest is in the abiotic drivers themselves or in other processes that structure plant communities, a comprehensive understanding of the influence of abiotic conditions is critical.

Research concerning abiotic enrichment (Clark & Tilman, 2008), the stress‐gradient hypothesis (Maestre, Callaway, Valladares, & Lortie, 2009), the available energy (AE) hypothesis (Lundholm, 2009), and others (Burton, Mladenoff, Clayton, & Forrester, 2011; McEwan & Muller, 2011) relate community patterns to the amount or level of key abiotic factors, such as the concentration of a soil nutrient. In these situations, abiotic conditions at a site could be measured in terms of the mean level of each important factor, and the species richness at that site results in part from how many species have these mean conditions within their niche (Scheffer & van Nes, 2006). If the total number of species able to tolerate different abiotic conditions varies in a systematic way (Myers & Harms, 2009), such that there is a tendency toward greater richness as conditions become more favorable, then we would expect to see a correlation between the mean abiotic conditions and species richness. Relationships between mean conditions and richness may be further influenced by relative fitness differences among competitors for different levels of a limiting resource (Clark & Tilman, 2008), or by interactions with dispersal ability leading to mass effects (Mouquet & Loreau, 2003; Shmida & Wilson, 1985).

Abiotic heterogeneity—as measured by the spatial variance or coefficient of variation of abiotic variables—can also be related to patterns of species richness (Stein, Gerstner, & Kreft, 2014). Relationships between species diversity and abiotic heterogeneity are commonly referred to as heterogeneity–diversity relationships (HDRs; Lundholm, 2009), terminology that we adopt throughout this paper. We might expect plant richness to increase with abiotic heterogeneity if communities are structured by niche differences (Chu & Adler, 2015; Kneitel & Chase, 2004; McKane et al., 2002). HDRs can also arise in other ways, such as when abiotic heterogeneity promotes microbial diversity which in turn increases plant species diversity (van der Heijden et al., 1998), or if sampling units encompass a wide range of abiotic conditions, for example, across habitat types with different regional species pools (Davies et al., 2005).

There is no a priori reason that both the AE and HDR hypotheses cannot act simultaneously (HilleRisLambers et al., 2012; Kerr & Packer, 1997; Kreft & Jetz, 2007). When they do, both the mean and the variance in abiotic conditions will influence plant diversity. Observational studies can be extremely helpful for confronting the complexity inherent in the sheer number of distinct ways that abiotic conditions can scale up to community patterns. Using observational data from a natural resource agency, we show how characterizing both the mean and heterogeneity in abiotic conditions simultaneously is a powerful way to identify potential mechanisms structuring herbaceous plants across spatial scales, seasons, and forest community types. This type of analysis can be a productive precursor to future experiments that pin down mechanism, particularly in understudied systems.

The current study addresses the following three gaps common in plant community ecology: (1) plants respond to an array of abiotic factors (Pausas & Austin, 2001), but many studies only include one or a few factors (Stevens & Carson, 2002). (2) When studies do include more than one abiotic factor, they are usually concerned with the effect of either abiotic mean conditions or abiotic heterogeneity on community patterns (e.g., Burton et al., 2011; Tamme, Hiiesalu, Laanisto, Szava‐Kovats, & Pärtel, 2010; but see Richard, Bernhardt, & Bell, 2000), or they treat mean conditions and heterogeneity as alternative hypotheses (Lundholm, 2009). And finally, (3) Abiotic drivers change across spatial and temporal scales (e.g., soil calcium with bedrock type [Bellemare, Motzkin, & Foster, 2005], or nitrogen with season, as in the vernal dam hypothesis [Tessier & Raynal, 2003]), but many studies consider only a single scale (Baer, Blair, Collins, & Knapp, 2004; Kreft & Jetz, 2007). We addressed points (1)–(2) through a statistical analysis of 29 herbaceous layer forb communities located across four common forest types found in the Allegheny plateau ecoregion, for which we have estimates of both the mean and spatial variance of 17 different abiotic factors (34 abiotic factor measurements). To explore point (3), we ran analyses across 2 spatial scales, repeated in spring and summer. Using these data, we built a suite of linear and linear mixed‐effects models to determine which abiotic factor measurements explained the most variation in species richness within and across different forest community types at two spatial scales in spring compared to summer.

## 2 METHODS

### 2.1 Study system

**Figure 1:**
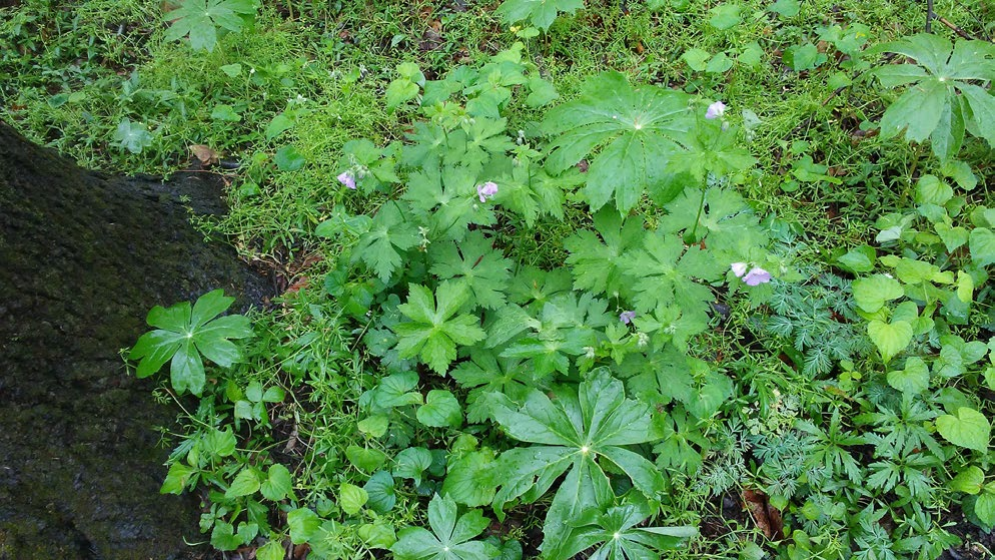
Example forb community in a forest understory located in Northeast Ohio. Species pictured include *Cardamine concatenata*, *Dicentra* sp., *Geranium maculatum*, *Floerkea proserpinacoides, Podophyllum peltatum*, and *Viola* sp.

Forbs—flowering, nongrass herbaceous plants—are an important yet understudied component of forest ecosystems (Gilliam, 2014; Figure 1). Often both common and diverse, forbs contribute to ecosystem services like nutrient cycling (Gilliam, 2007), and serve as nectaries and hosts for pollinators (Bess, 2005; Davis & Cipollini, 2014; Hanula, Ulyshen, & Horn, 2016). They also account for a large proportion of plant biodiversity in temperate forests. For example, most spring ephemerals (species that begin to bloom in early April, then die back and give way to a summer vegetation community at the end of May) are forbs, and therefore drive much of the compositional change in plant communities across seasons (Gilliam, 2014). There have been a multitude of studies assessing the connection between resource availability and herbaceous layer species richness in temperate forests (including forbs; e.g., Bellemare et al., 2005; Burton et al., 2011; Lundholm, 2009; McEwan & Muller, 2011; Peet, Palmquist, & Tessel, 2014). Recent studies have also highlighted the importance of biotic processes in forest forb communities, like dispersal limitation (Burton et al., 2011; Ehrlén & Eriksson, 2000; Flinn & Vellend, 2005; Pärtel, Szava‐Kovats, & Zobel, 2011) and the role of plant‐soil feedbacks (Burke, Klenkar, & Medeiros, 2018). Much less common, however, are studies examining the relationship between temperate forest forbs and abiotic heterogeneity.

### 2.2 Study area

Sampling was conducted in collaboration with a monitoring program implemented by Cleveland Metroparks (CMP). The sites used in our analysis are a subset of 100 sights chosen randomly using the Generalized Random Tessellation Stratified (GRTS) spatial sampling method at the start of the monitoring program in 2010 (Mack & Robison, 2010). At each site, a 400 m^2^ plot composed of four contiguous 100 m^2^ subplots (of any configuration) was placed to encompass an area with similar canopy species, and without any conspicuous inclusions (e.g., a vernal pool, or a drastic change in slope aspect). One plot with just two subplots was also included in our analysis.

**Figure 2:**
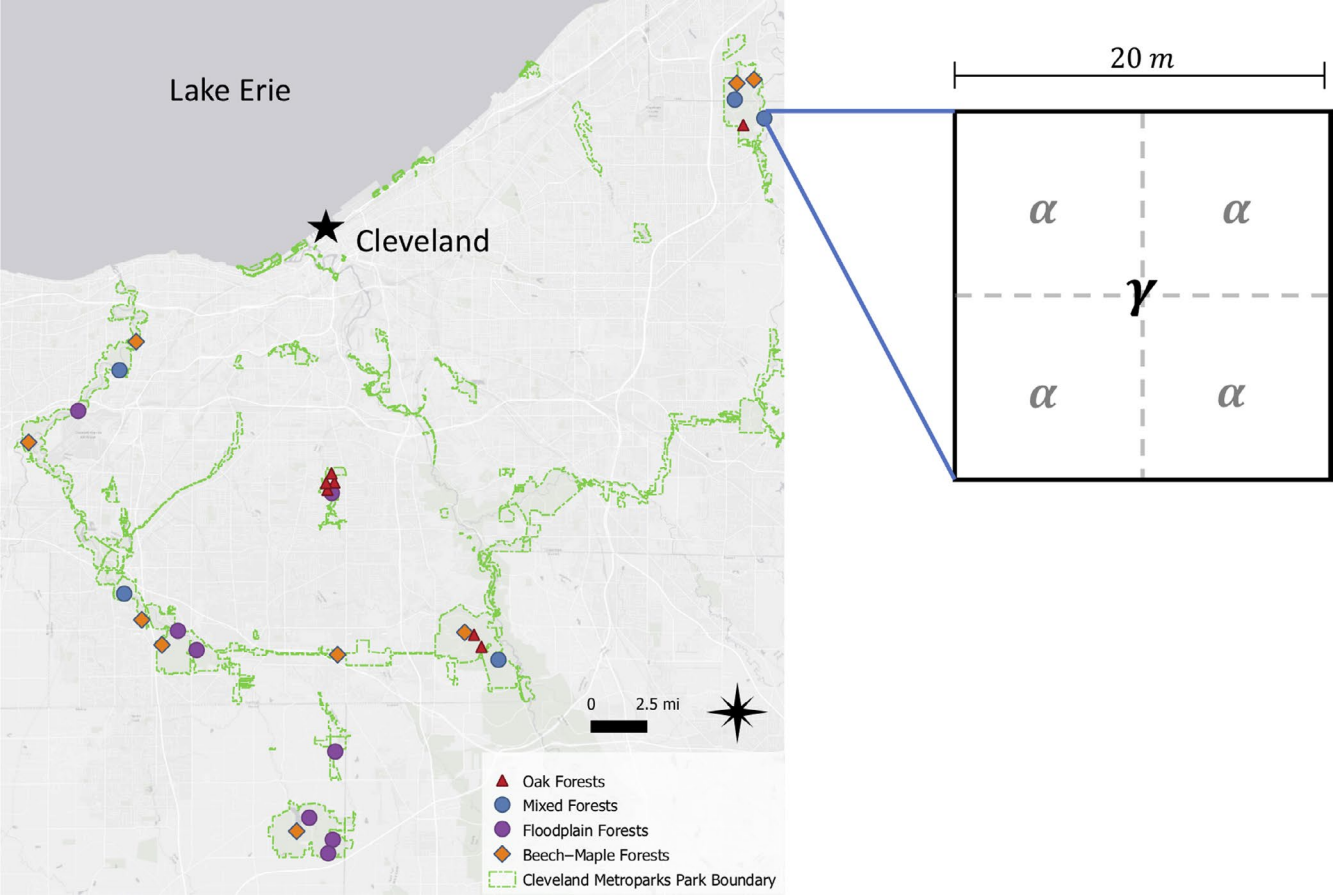
The distribution of sampled plots and the forest community types they represent within Cleveland Metropark's park boundaries (located around Cleveland, Ohio). Pictured alongside is an example of a single plot, with levels *γ* (whole plot) and *α* (subplots) used to denote at what scale measurements were taken

Spring sampling was conducted by a crew led by the first author (17 April–14 May), and summer sampling was conducted by two crews of CMP employees led by the second author (23 June–8 September). CMP's crews only sample in summer, so our spring sampling was done without the benefit of CMP's field staff. For this reason, visiting all 100 sites was not feasible in spring. As such, we selected 4–5 plots in each of 6 land holdings (29 plots total) that had at least one tree individual >60 diameter at breast height (DBH; to exclude early successional communities). In each land holding, we attempted to select plots representing the four terrestrial hardwood community types commonly found in Northeast Ohio: Beech–Maple, Floodplain, Mixed, and Oak forests (Eysenbach & Hausman, 2013), although this was not always possible (Figure 2).

### 2.3 Field sampling protocol

Sampling procedures were based on the North Carolina Vegetation Survey Method (Peet, Wentworth, & White, 1998). Data were collected on all plant species (including non‐natives) found growing to the height of the tallest herbaceous species off the forest floor, including tree seedlings, ferns, grasses, sedges, mosses, and forbs. As we did not directly control for functional and/or phylogenetic relatedness in our study, we limited our analyses to include only forb species because we were especially interested in spring ephemeral communities. Species cover was assessed in each subplot (*α*) and each plot (*γ*) using the Braun‐Blanquet method (Peet et al., 1998; Figure 2). Cover data were converted to presence–absence data to get a species count in each plot (plot‐level species richness, *γ*_S_) and subplot (subplot‐level species richness, *α*_S_). Sometimes, individuals of the same genus but different species (identifiable via unique characteristics, like smooth or hairy stems in the genus *Solidago*) were nevertheless unidentifiable to species at the time of sampling. These were identified as *Genus* sp. 1, 2, 3, etc., allowing us to accurately measure species richness without a confirmed species identification.

Approximately 340 g of soil taken from within 10 cm of the surface were collected with a soil probe at randomly chosen points throughout each subplot (8 plugs/subplot), providing subplot‐level composites for each abiotic factor *j*, *α_j_*. Undried soil samples were stored in Whirl‐Pak bags at room temperature for the sampling season, and then sent to A&L Great Lakes Laboratories (Fort Wayne, Indiana, USA) where samples were dried, ground, sieved and then analyzed for a suite of variables known to affect plant performance: organic matter (%), phosphorus (Bray 1 parts‐per‐million [ppm]), pH, cation exchange capacity (meq/100 g), nitrogen (% total), carbon (%), and the carbon:nitrogen ratio. Potassium, magnesium, and calcium were also measured, as both absolute quantities (e.g., in ppm), and relative to one another (e.g., % base saturation; Table 1).

**Table 1:** Minimum and maximum measured values of all predictor variables *α_j_* for abiotic factor *j* across all subplots *α*

Abiotic factors (units)	Notation	Sampled range (*α_j_*)	
		Spring	Summer
Organic matter (%)	*α*_OM_	1.7–14.6	1.7–31.5
Phosphorus (Bray 1 ppm)	*α*_P_	1.0–191.0	1.0–22.0
Potassium (ppm)	*α*_Kppm_	41.0–162.0	23.0–153.0
Magnesium (ppm)	*α*_Mgppm_	30.0–300.0	30.0–345.0
Calcium (ppm)	*α*_Cappm_	100.0–3,400.0	50.0–2,800.0
pH	*α*_pH_	3.5–8.0	3.6–7.2
Cation exchange capacity (meq/100 g)	*α*_CEC_	3.4–26.8	1.9–24.9
Potassium base saturation (%)	*α*_K_	0.6–7.4	0.7–5.7
Magnesium base saturation (%)	*α*_Mg_	1.0–27.4	1.0–24.6
Calcium base saturation (%)	*α*_Ca_	2.2–91.9	2.0–89.3
Carbon (%)	*α*_C_	1.0–8.5	1.0–18.3
Nitrogen (% total)	*α*_N_	0.0–0.6	0.0–0.8
Carbon to nitrogen ratio	*α*_C:N_	8.9–36.9	10.3–29.0
Light*1.04 (%)	*α*_light_	62.0–95.7	0.2–23.2
Litter depth (cm)	*α*_ld_	0.0–11.0	0.0–5.1
Organic layer depth (cm)	*α*_od_	0.0–3.5	–
Restrictive layer depth (cm)	*α*_rd_	9.0–101.0	9.0–101.0

Note

Soil chemistry variables (organic matter–C:N ratio) represent a composite of soil taken across a subplot. Remaining variables (light–restrictive layer depth) were measured from the center of each subplot.

In the center of each subplot, leaf litter and organic layer depth were measured to the nearest 10th of a centimeter, and light data were collected using a densiometer. Restrictive layer depth was also measured in the center of each subplot using a tile probe (Table 1). Organic layer depth in summer was extremely zero‐inflated and was excluded from the analysis.

### 2.4 Seasonal designations

Each plant was designated a spring or summer species based on the date of up to three flowering specimens collected since 1990 found digitized in the Ohio State Herbarium (Museum of Biological Diversity Herbarium, The Ohio State University, 2016; Table 2). Observed flowering times were also recorded in the field by the first author and used to corroborate and supplement dates collected online. Spring and summer species had an average flowering date before and after June 1, respectively (except *Allium tricoccum*, which was designated as a spring species since it loses its spring vegetative parts before flowering and senescing in June [Vasseur & Gagnon, 1994]). Only designated spring species were used in the spring sample, whereas all identifiable plants were used in the summer sample, and thus often included spring ephemeral species that had not yet senesced, especially early in the summer season. We chose this approach because spring species that persist into summer have fully emerged, while summer species that start to appear in spring have not. Species that were only identifiable to genus were included in the analysis and designated a season based on the natural history of their taxonomic group (e.g., violets only identifiable to *Viola* sp. were designated as spring species).

**Table 2:** List of forb species identified in our study

Spring species	Summer species
*Actaea pachypoda*	*Actaea pachypoda*
*Alliaria petiolata*	*Ageratina altissima var*. *altissima*
*Allium canadense*	*Agrimonia gryposepala*
*Allium tricoccum*	*Agrimonia parviflora*
*Anemone quinquefolia*	*Agrimonia pubescens*
*Arisaema dracontium*	*Agrimonia* sp.
*Arisaema triphyllum*	*Alliaria petiolata*
*Asarum canadense*	*Allium canadense*
*Barbarea verna*	*Allium tricoccum*
*Camassia scilloides*	*Amphicarpaea bracteata*
*Cardamine concatenata*	*Apocynum cannabinum*
*Cardamine diphylla*	*Arctium lappa*
*Cardamine douglassii*	*Arisaema dracontium*
*Cardamine bulbosa*	*Arisaema triphyllum*
*Caulophyllum thalictroides*	*Asarum canadense*
*Claytonia virginica*	*Bidens* sp.
*Conopholis americana*	*Blephilia hirsuta*
*Dicentra canadensis*	*Caulophyllum thalictroides*
*Dicentra cucullaria*	*Cerastium fontanum* ssp. *vulgare*
*Duchesnea indica*	*Circaea lutetiana*
*Enemion biternatum*	*Clematis virginiana*
*Erigenia bulbosa*	*Collinsonia canadensis*
*Erythronium americanum*	*Conium maculatum*
*Floerkea proserpinacoides*	*Conopholis americana*
*Fragaria virginiana*	*Cryptotaenia canadensis*
*Galium aparine*	*Desmodium glutinosum*
*Geranium maculatum*	*Doellingeria umbellata*
*Geum vernum*	*Duchesnea indica*
*Glechoma hederacea*	*Epifagus virginiana*
*Hepatica nobilis*	*Epilobium coloratum*
*Hesperis matronalis*	*Epilobium *sp.
*Houstonia caerulea*	*Epipactis helleborine*
*Hydrophyllum virginianum*	*Equisetum hyemale*
*Lilium *sp.	*Erechtites hieraciifolius*
*Luzula multiflora*	*Erigeron annuus*
*Maianthemum canadense*	*Eurybia macrophylla*
*Maianthemum racemosum*	*Euthamia graminifolia*
*Mertensia virginica*	*Eutrochium maculatum*
*Mitchella repens*	*Eutrochium purpureum*
*Mitella diphylla*	*Fragaria vesca*
*Nabalus *sp.	*Galium aparine*
*Osmorhiza claytonii*	*Galium concinnum*
*Osmorhiza longistylis*	*Galium* sp.
*Oxalis stricta*	*Galium triflorum*
*Packera aurea*	*Geranium maculatum*
*Packera obovata*	*Geum canadense*
*Panax trifolius*	*Geum* sp.
*Phlox divaricata*	*Glechoma hederacea*
*Podophyllum peltatum*	*Hackelia virginiana*
*Polemonium reptans*	*Heliopsis helianthoides*
*Polygonatum biflorum*	*Hepatica nobilis*
*Polygonatum pubescens*	*Heracleum maximum*
*Potentilla simplex*	*Hesperis matronalis*
*Prosartes lanuginosa*	*Houstonia caerulea*
*Ranunculus abortivus*	*Hydrophyllum canadense*
*Ranunculus fascicularis*	*Hydrophyllum virginianum*
*Ranunculus ficaria*	*Hypericum perforatum*
*Ranunculus hispidus*	*Impatiens capensis*
*Ranunculus recurvatus*	*Impatiens pallida*
*Ranunculus repens*	*Impatiens* sp.
*Ranunculus* sp.	*Iris* sp.
*Sanguinaria canadensis*	*Jeffersonia diphylla*
*Symplocarpus foetidus*	*Juncus tenuis*
*Taraxacum officinale*	*Lactuca* sp.
*Tiarella cordifolia*	*Laportea canadensis*
*Thalictrum thalictroides*	*Lobelia* sp.
*Trillium erectum*	*Luzula acuminata*
*Trillium grandiflorum*	*Lycopus* sp.
*Trillium sessile*	*Lysimachia ciliata*
*Trillium *sp.	*Lysimachia nummularia*
*Tussilago farfara*	*Maianthemum canadense*
*Unknown forb*	*Maianthemum racemosum*
*Uvularia sessilifolia*	*Mitchella repens*
*Veronica officinalis*	*Mitella diphylla*
*Viola blanda*	*Monarda clinopodia*
*Viola canadensis*	*Monarda fistulosa*
*Viola pubescens*	*Monotropa uniflora*
*Viola rostrata*	*Myosotis scorpioides*
*Viola sororia*	*Nabalus* sp.
*Viola* sp.	*Osmorhiza claytonii*
*Viola striata*	*Osmorhiza longistylis*
*Zizia aptera*	*Oxalis stricta*
*Zizia aurea*	*Packera obovata*
	*Persicaria hydropiperoides*
	*Persicaria maculosa*
	*Persicaria sagittata*
	*Persicaria* sp.
	*Persicaria virginiana*
	*Phytolacca americana*
	*Pilea pumila*
	*Plantago rugelii*
	*Plantago* sp.
	*Podophyllum peltatum*
	*Polemonium reptans*
	*Polygonatum biflorum*
	*Polygonatum pubescens*
	*Potentilla simplex*
	*Prosartes lanuginosa*
	*Prunella vulgaris*
	*Ranunculus abortivus*
	*Ranunculus acris*
	*Ranunculus hispidus*
	*Ranunculus recurvatus*
	*Ranunculus* sp.
	*Rubus hispidus*
	*Rudbeckia laciniata*
	*Rumex obtusifolius*
	*Rumex* sp.
	*Sanguinaria canadensis*
	*Sanicula odorata*
	*Sanicula* sp.
	*Scutellaria lateriflora*
	*Sedum ternatum*
	*Sisyrinchium* sp.
	*Solidago caesia*
	*Solidago flexicaulis*
	*Solidago gigantea*
	*Solidago patula*
	*Solidago rugosa*
	*Solidago* sp.
	*Symphyotrichum cordifolium*
	*Symphyotrichum lanceolatum*
	*Symphyotrichum lateriflorum*
	*Symphyotrichum *sp.
	*Symplocarpus foetidus*
	*Taraxacum officinale*
	*Thalictrum dasycarpum*
	*Thalictrum pubescens*
	*Thalictrum thalictroides*
	*Thaspium barbinode*
	*Tiarella cordifolia*
	*Trifolium repens*
	*Trillium *sp.
	*Tussilago farfara*
	*Verbena urticifolia*
	*Verbesina alternifolia*
	*Veronica officinalis*
	*Veronica serpyllifolia*
	*Viola pubescens*
	*Viola* sp.
	*Viola striata*
	*Zizia aurea*

Note

The spring species list does not include species with an average flowering date >June 1, as these were not included in our statistical analysis. The summer species list includes all identifiable species, including persisting spring species.

### 2.5 Community patterns and abiotic measurements

Species presence data were recorded for each plot (to compute plot‐level species richness, *γ*_S_) and subplot (to compute subplot‐level species richness, *α*_S_; Figure 2). As all abiotic factors were measured at the subplot level, we were able to assess the magnitude of abiotic conditions at both the subplot level and, by averaging across subplot‐level values, at the plot level (*α_j_* and *ᾱ_j_*, respectively). Abiotic heterogeneity could only be assessed at the plot level and was measured as the coefficient of variation (CV) across subplot‐level values within a plot (cv(*α_j_*)) (Figure 2). Abiotic factors were measured in units of very different magnitude (Table 1). To facilitate comparison between linear models, all abiotic predictor variables (i.e., the raw data taken at the subplot‐level, the plot‐level means as measured via the raw data, and the plot‐level coefficient of variation as measured via the raw data) were first normalized, if strongly skewed, via a log or square root transformation. All measurements were then standardized (z‐transformed) to have mean of 0 and a variance of 1.

### 2.6 Analysis

#### 2.6.1 Statistical modeling and model selection

**Figure 3:**
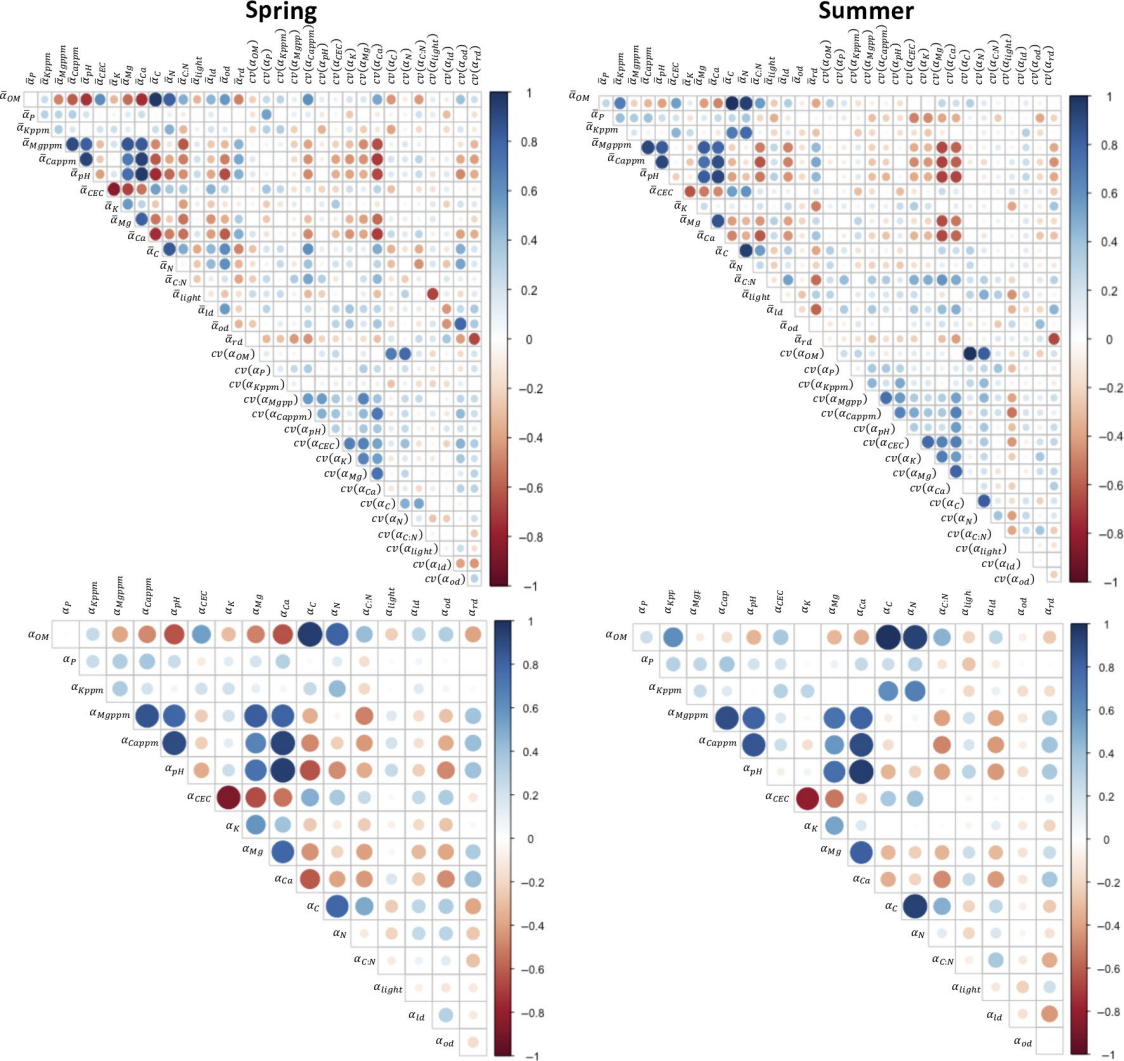
Correlation structure across abiotic factor measurements taken in spring and summer

Our first goal was to identify the best statistical models of plot‐level and subplot‐level species richness (*γ*_S_ and *α_S_*, respectively) via model selection. Our general approach was to begin with a full model for species richness, *γ*_S_, that included the plot‐level means of all 17 abiotic factors (*ᾱ_j_*) and then conduct model selection to determine the best predictors of species richness. Except where noted, all model selection was done using the dredge function in the R package MuMIn (Bartoń, 2018), wherein subsets of a full model were ranked according to their AIC_C_ score (Hurvich & Tsai, 1989). Since there was collinearity between multiple abiotic measurements (Figure 3), we restricted the dredge so that any pairs of abiotic terms with >40% correlation would not be included together in any model subsets. After each dredge, models with an AIC_C_ score within 2.0 of the best model's AIC_C_ were retained. Diagnostics were then run on each retained model to check for outliers and normally distributed residuals. If present, we removed the outlier and/or log‐ or square root‐transformed the response variable to meet the assumption of normally distributed residuals, then re‐dredged the full model. If there were outliers and non‐normal residuals, normality was corrected first.

We next repeated this procedure with a full model that included the plot‐level heterogeneities (cv(*α_j_*)) instead. Once the best mean‐based and CV‐based models were attained, we combined the surviving terms from each into a third full model and conducted model selection again, to see whether the combined model would better predict species richness compared to the best models based on abiotic mean or CV alone. To facilitate comparison of AIC_C_ scores, we removed the same outliers from all full models within a season (e.g., if a plot was an outlier in the mean model, it was also removed from the CV model). We identified the best statistical models for subplot‐level species richness (*α_S_*) in the same way, using sampled subplot‐level conditions (*α_j_*) as abiotic predictors.

#### 2.6.2 Assessing different model structures

The procedure described in the preceding paragraph was repeated for several classes of “full model,” including linear models that treated all plots as independent sampling units, and linear mixed‐effects models that allowed plot or subplots in different forest types to differ in their slope and/or intercept (Table 3). In other words, linear mixed‐effects models were used to account for the nonindependence of plots found in similar forest communities (step B in Table 3).

**Table 3:** Stepwise protocol used to generate candidate best models for species richness

Step	Model type	Question	Modeling plot‐level richness (*γ*_S_)
A: across plots	Linear	Can abiotic factors explain species richness across plots?	*Mean model A*: full model included the mean of all abiotic factors as predictors. *CV model A*: full model included the variability of all abiotic factors as predictors.
B: unconditional means	Linear mixed‐effects	Are observations correlated with forest community type?	*Unconditional model B*: contained no abiotic predictors, only a grouping factor (e.g., plots in forests).
C–D	Linear mixed‐effects	Can abiotic factors explain patterns across and/or within forests?	Repeated step A, with forest type added as a grouping factor in the various full models
C1: across plots accounting for forest	Linear mixed‐effects with variable intercepts	Do abiotic factors explain changes in community patterns across forest types?	*C1 models*: keeping slopes constant, intercepts varied by forest community type.
C2: within forests	Linear mixed‐effects with variable slopes	Do abiotic factors explain changes in community patterns within forest types?	*C2 models*: slopes varied by forest type with a single intercept.
D1: across plots accounting for forest, and within forests	C1 with variable slopes	Do the same or different abiotic factors explain patterns within forest types?	*D1 models*: allowed slopes to vary without forcing intercepts through a fixed point.
D2: within forests, and across plots accounting for forest	C2 with variable intercepts	Do the same or different abiotic factors explain patterns across plots and forests?	*D2 models*: allowed intercepts to vary alongside slopes.

Each class of model allowed us to answer a different question about the richness patterns we observed. Linear models (step A in Table 3) describe the relationship between species richness and abiotic factors across all plots, regardless of forest community type. Linear mixed‐effects (LME) models (steps C‐D in Table 3) describe the relationship between species richness and abiotic factors within forest types. For example, a variable intercepts model (step C1) suggests that mean richness across forests varies but the overall relationship with the abiotic factor does not. A variable slopes model (step C2) suggests that the overall relationship with an abiotic factor depends on forest community type. In steps D1 and D2, best models from C1 and C2 were modified to allow slopes and intercepts to vary simultaneously.

We did not build linear models for subplot‐level richness because of the spatial proximity of subplots within plots. Instead, we compared two types of LME models: one with subplots grouped by plots, and another with subplots grouped by plots nested in forest type.

Models were screened for variable slopes in an iterative fashion (i.e., separate models were built to cycle through each abiotic measurement separately, instead of dredging a full model). In models with variable slopes and intercepts (e.g., step D1 in Table 3), we did not force slopes and intercepts to vary according to a particular abiotic factor. This allowed us to see whether a different abiotic measurement was important within different forest community types, compared to those driving patterns across plots and/or forest types. To make sure this practice was not obscuring a best model, we also built expanded models where any abiotic measurement associated with variable slopes was also added to the suite of best predictors identified in previous steps. In all LME models, a diagonal positive definite matrix was used to specify the variable slopes part of the model to control for any covariance between intercepts and slopes.

All linear models were fit using ordinary least squares. All LME models were fit using maximum likelihood in the lme function in the R package nlme (Pinheiro, Bates, DebRoy, Sarkar, & R Core Team, 2018). The variation explained by LME models is given in terms of marginal‐ and conditional‐*r* ^2^ values (Johnson, 2014). The marginal‐*r* ^2^ value is the variance explained in species richness across plots—for example, the single intercept and slope for each predictor that describes its effect on species richness without accounting for forest type. The conditional‐*r* ^2^ value in the LME model is the marginal‐*r* ^2^ plus the variance explained by the intercepts and/or slopes that vary by forest type.

Each step in Table 3 yielded best candidate models describing how richness is related to mean abiotic conditions, abiotic heterogeneity, or both (except B, which considered the null model including no abiotic predictor variables). From among these, AIC_C_ scores and the amount of variance explained across study levels were used to choose a suite of overall best models for each season. All analyses were conducted in R (R Foundation for Statistical Computing).

### 2.7 Variance partitioning

After assembling a suite of best models, variance partitioning was used to determine whether more variance in plot‐level species richness could be attributed to mean abiotic conditions or to abiotic heterogeneity. This allows a higher‐level synthesis of the results from our series of fitted models. For each season, the varpart function in the R package vegan (Oksanen et al., 2018) was used to partition the variance in species richness explained into the portion attributed to mean abiotic conditions, the portion attributed to the heterogeneity in abiotic conditions, the portion attributed to both (i.e., indistinguishable due to correlation), and the portion that remained unexplained by our abiotic measurements.

Using the abiotic predictors from the subset of best model(s) from the statistical analysis, we did variance partitioning at two different levels: once using all plots and excluding abiotic predictors whose slopes varied with forest community type, and once for plots within each forest type and including abiotic factor predictors whose slopes varied depending on forest. In the latter analysis, we only partitioned variance between two abiotic predictors at a time since sample size was low within forests (*n* = 6, 9, 5, and 9 for Beech–Maple, Floodplain, Mixed, and Oak forests, respectively). We then compared results across all pairwise combinations of abiotic predictors, but retained only those pairs that had the smallest amount of unexplained variance to use in our analysis.

## 3 RESULTS

### 3.1 Statistical modeling

**Figure 4:**
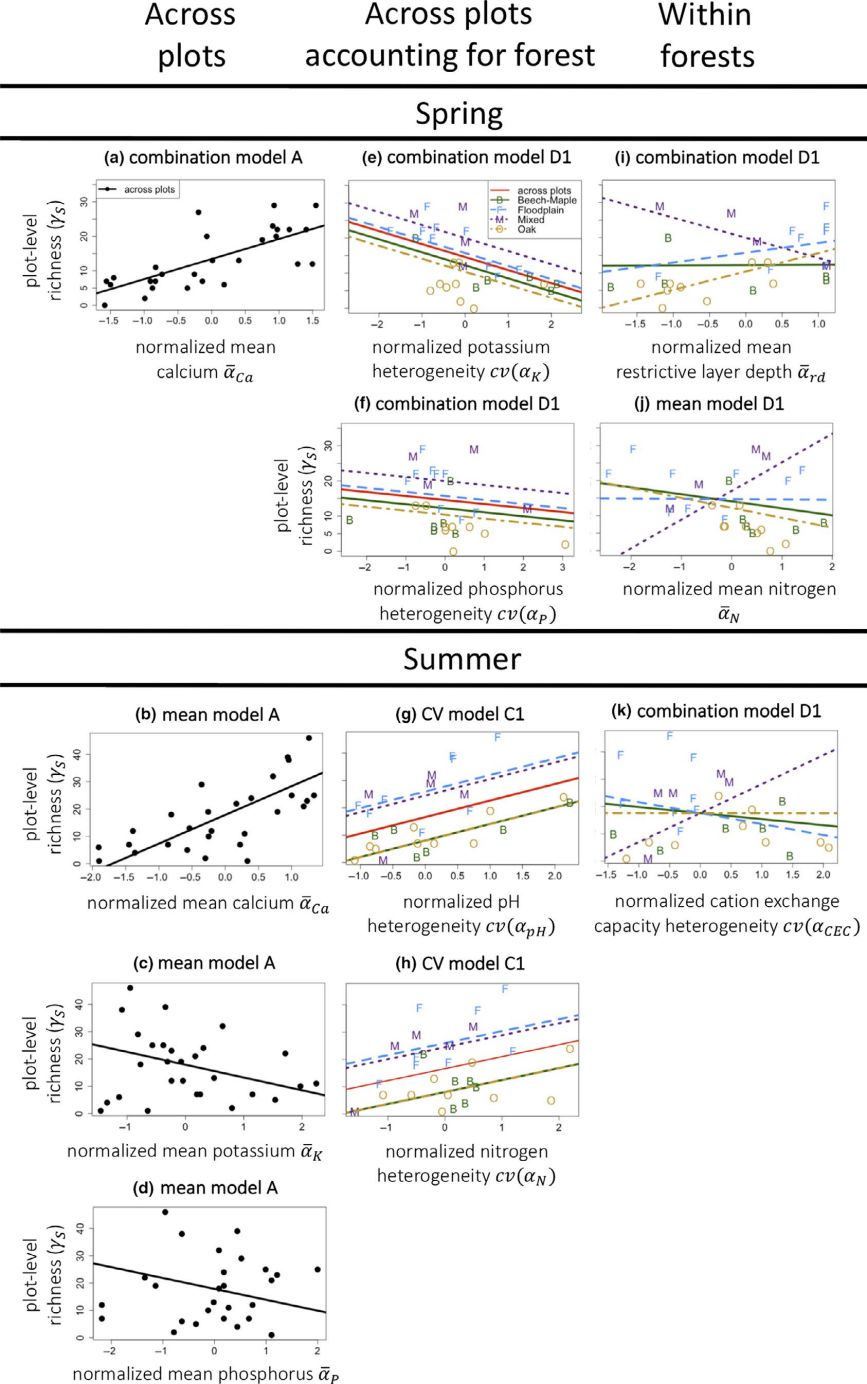
Selected trends in plot‐level richness (*γ*_S_). Plotted relationships correspond to models listed in Table 4 (and described in Table 3)

Mean abiotic conditions and heterogeneity in abiotic conditions across multiple factors together explained substantial variation in plot‐level forb richness in both seasons (Table 4). For example, across plots (i.e., from step A in Table 3), plot‐level richness increased with mean calcium availability in both seasons (Figure 4a,b) and decreased with mean phosphorus and potassium availability in summer (Figure 4c,d). Across plots and after accounting for forest type (steps C–D), we found negative heterogeneity–diversity relationships (HDRs) in spring (Figure 4e,f), but positive HDRs in summer (Figure 4g,h). Within forest types, trends in plot‐level richness changed according to mean abiotic conditions in spring (Figure 4i,j), but heterogeneity of different abiotic factors in summer (Figure 4k).

**Table 4:** Selected models explaining plot‐ and subplot‐level species richness

Community pattern	Season	Best model(s)	Predictors	∆AICc	*r* ^2^ (%)	Conditional‐*r* ^2^ (%)
Plot‐level richness (*γ*_S_)	Spring	Combination model D1	$\text{cv}\left(\alpha_{P}\right)+\text{cv}\left(\alpha_{K}\right)+\left(1|\text{forest}\right)+\left(\left.{\bar{\alpha }}_{\text{rd}}\right|\text{forest}\right)$	0.0	25	83
		Combination model A	${\bar{\alpha }}_{\text{Ca}}+\text{cv}\left(\alpha_{P}\right)$	3.9	56	–
		Mean model D1	${\bar{\alpha }}_{\text{Ca}}+\left(1|\text{forest}\right)+\left(\left.{\bar{\alpha }}_{N}\right|\text{forest}\right)$	7.6	36	75
	Summer	Mean model A	${\bar{\alpha }}_{\text{Ca}}+{\bar{\alpha }}_{K}+{\bar{\alpha }}_{P}$	0.0	61	–
		Combination model D1	${\bar{\alpha }}_{\text{Ca}}+{\bar{\alpha }}_{K}+{\bar{\alpha }}_{P}+\text{cv}\left(\alpha_{\text{pH}}\right)+\left(1|\text{forest}\right)+\left(\text{cv}\left(\alpha_{\text{CEC}}\right)-1|\text{forest}\right)$	3.3	59	81
		CV model C1	$\text{cv}\left(\alpha_{\text{pH}}\right)+\text{cv}\left(\alpha_{N}\right)+\left(1|\text{forest}\right)$	8.0	28	74
Subplot‐level richness $\left(\sqrt{\alpha_{S}}\right)$	Spring	Plot model C1	$\alpha_{\text{Ca}}+\left(1|\text{plot}\right)$	0.0	11	88
	Summer	Plot model D1	$\alpha_{\text{Ca}}+\alpha_{K}+\alpha_{\text{OM}}+\left(1|\text{plot}\right)+\left(\alpha_{K}|\text{plot}\right)$	0.0	41	88

Note

The notation “$\left|\text{forest}\right.$” indicates what grouping factor was used in linear mixed‐effects models—that is, either forest, plot, or plots in forests (i.e., “$\left|\text{forest}/\text{plot}\right.$”). The value listed in the *r* ^2^ column is either the standard multiple‐ or adjusted‐*r* ^2^ in linear models with one predictor or multiple predictors, respectively, or the marginal‐*r* ^2^ for linear mixed‐effects models.

**Figure 5:**
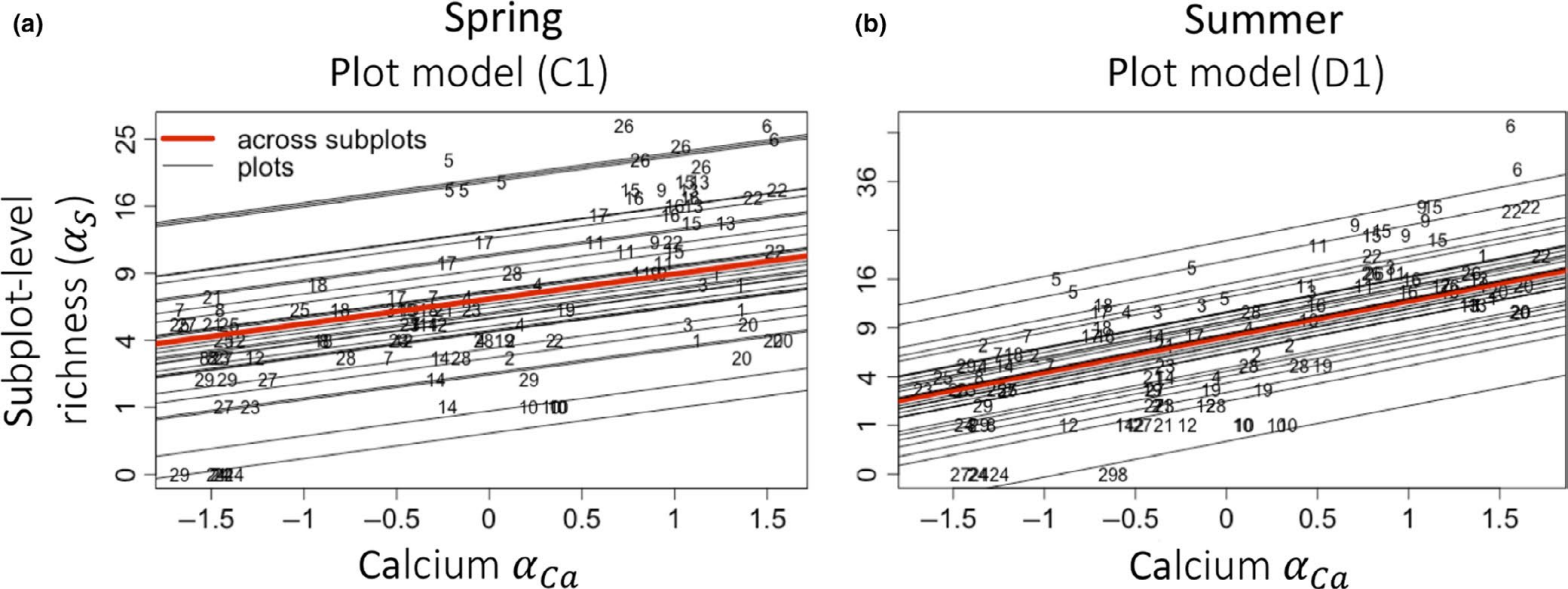
Selected trends in subplot‐level species richness (*α*_S_). Plotted relationships correspond to models listed in Table 4. Numbers indicate to which plot each subplot belonged. The *x*‐axes for all abiotic predictors have been normalized for comparison, and community pattern response variables that were log‐ or square root‐transformed for analysis have been plotted with the original values on the *y*‐axis

After accounting for plot, subplot‐level richness increased across subplots with increasing mean subplot‐level calcium in both seasons, but this relationship was much stronger in summer (slope = 0.73) compared to spring (slope = 0.37; Figure 5a,b).

### 3.2 Variance partitioning

**Figure 6:**
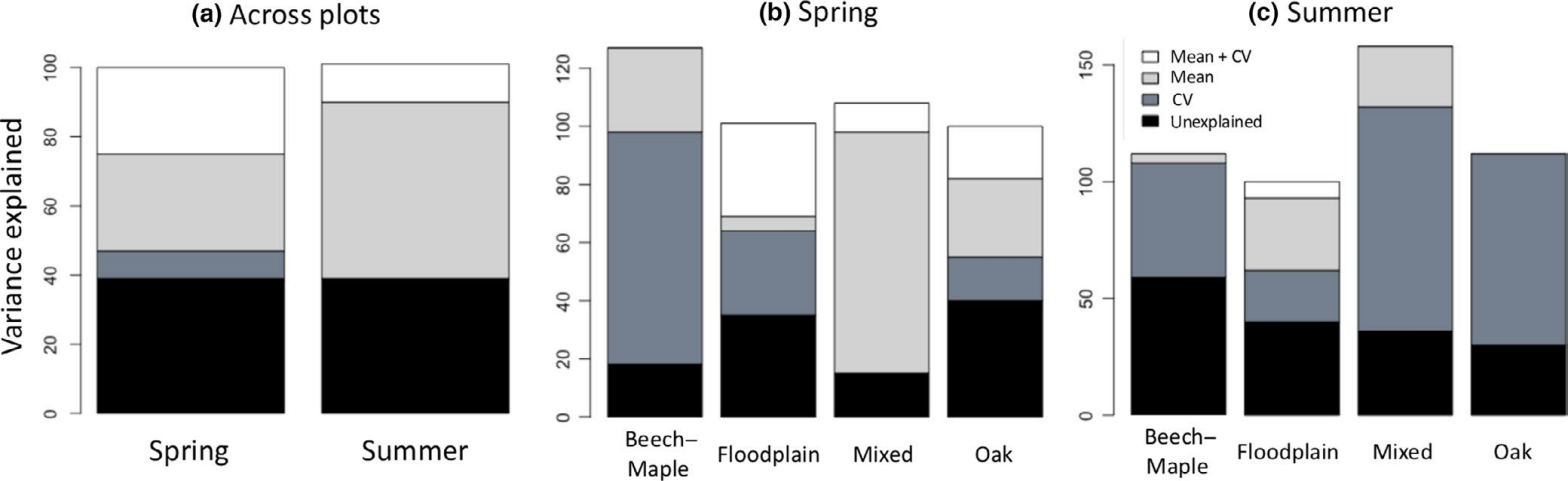
Proportion of variance explained in plot‐level species richness, *γ*_S_, partitioned between mean abiotic conditions (light gray), abiotic heterogeneity (CV, in dark blue), both (mean + CV in white), and neither (unexplained in black). To partition variance explained across all plots before accounting for forest type (a), we excluded abiotic factor predictors that changed trends across forest community types (e.g., abiotic factors in column 3 of Figure 4). To partition variance explained within forest community types (b and c), we partitioned the variance explained in plot‐level species richness in each forest and included abiotic factor predictors that changed trends across forest community type

Across all plots, most of the variability in plot‐level species richness was attributed to mean abiotic conditions in spring, and in summer this trend became even more pronounced in that no variability could be attributed to abiotic heterogeneity alone (Figure 6a). Within most forest community types, however, more variability in species richness was attributed to abiotic heterogeneity than to mean abiotic conditions, and this was true for both seasons (Table 5, Figure 6b,c).

**Table 5:** Pairs of abiotic measurements within each forest type that resulted in the lowest amount of unexplained variation in plot‐level species richness after partitioning variance due to the abiotic mean, the abiotic CV, both, or neither

	Spring		Summer	
	Mean	Heterogeneity	Mean	Heterogeneity
BM	${\bar{\alpha }}_{N}$	$\text{cv}\left(\alpha_{K}\right)$	${\bar{\alpha }}_{P}$	$\text{cv}\left(\alpha_{\text{pH}}\right)$
FP	${\bar{\alpha }}_{\text{rd}}$	$\text{cv}\left(\alpha_{K}\right)$	${\bar{\alpha }}_{K}$	$\text{cv}\left(\alpha_{\text{pH}}\right)$
M	${\bar{\alpha }}_{N}$	–	${\bar{\alpha }}_{\text{Ca}}$	$\text{cv}\left(\alpha_{\text{CEC}}\right)$
OAK	${\bar{\alpha }}_{\text{Ca}}$	$\text{cv}\left(\alpha_{P}\right)$	${\bar{\alpha }}_{K}$	$\text{cv}\left(\alpha_{\text{pH}}\right)$

Note

Light gray and dark blue bars in Figure 6b,c illustrate the proportion of variation explained by the mean and CV, respectively, in Beech–Maple (BM), Floodplain (FP), Mixed (M), and Oak forests. N, Ca, K, and P refer to elements by their atomic symbols.

Abbreviations: CEC, cation exchange capacity; rd, restrictive layer depth.

## 4 DISCUSSION

In spring, 50% of the variation in forb species richness was explained by forest community type alone, with no measured abiotic predictors included in the linear mixed‐effects models. In summer, only 30% of the variation in forb species richness was explained by forest community type, likely because Floodplain and Mixed forests, as well as Oak and Beech–Maple forests, had similarly sized species pools in summer. In other words, mean richness varied in a predictable way across forest community types in spring, and somewhat less so in summer. Differences in the mean number of species could be due to differences in the size of a shared species pool (e.g., if certain forest types include additional species in an otherwise shared pool), or to the presence of a unique species' pools for each forest community type. Notably, the AICc score of more complicated models including abiotic predictors were always much lower than the AICc scores of the null model including only forest community type (∆AIC_C_ = 15.9 in spring and ∆AIC_C_ = 16.2 in summer). A lower AICc score compared to the null model indicates that the additional variation in forb species richness patterns explained after adding abiotic predictors, for example, the intercept(s) and slope(s) of the models shown in Table 4 are not simply due to a more complex model, but actually reflect additional variation explained by the measured abiotic factors after accounting for differences in mean richness across forest types. We review which predictors best explained forb species richness and offer some brief interpretation of our results based on when we found support for the AE and HDR hypotheses.

### 4.1 Summary

Our results confirm that patterns of forb richness depend on both mean abiotic conditions and abiotic heterogeneity (Table 4, Figure 4). In spring, including both the mean and heterogeneity of different abiotic factors resulted in the best overall model and explained the most variation in plot‐level richness (Table 4), giving simultaneous support for the presence of heterogeneity–diversity relationships (HDRs) and the available energy (AE) hypothesis.

In summer, the mean model was the most supported according to its AIC_C_ score, but the heterogeneity model and the combined model explained more variance (Table 4). We suspect that the heterogeneity and combined models have inflated AIC_C_ scores because in summer, the penalty for dividing forest types into 4 categories was not justified given that Mixed and Floodplain forests had the same mean species richness, as did Oak and Beech–Maple forests. Since there was little correlation between means and CVs of the abiotic measurements included in the summer models (Figure 3), we think the explanatory power of the combined model is biologically meaningful and not simply due to collinearity. We therefore interpret our results as evidence that summer plot‐level richness is also determined by both mean abiotic conditions and their heterogeneity.

Because our analysis makes use of an observational dataset, we cannot isolate the mechanisms underlying our results. Nevertheless, our findings are suggestive of which mechanisms are likely to be structuring community patterns at different study levels. Below, we highlight a few intriguing results and discuss possible mechanisms that might explain them.

### 4.2 Positive heterogeneity–diversity relationships: pH and nitrogen heterogeneity in summer

In summer, richness increased with increasing pH and nitrogen heterogeneity: both positive HDRs (Figure 4g,h). This suggests that assemblages of summer species may differentiate along pH and nitrogen niche axes, while the lack of a similar HDR in spring suggests that assemblages of spring species do not. It is also possible that the spatial structure in pH and nitrogen changed seasonally such that HDRs occurred at a different scale in spring than summer, allowing only the summer relationship to be detected by our sampling scheme.

Many herbaceous forbs found in these forests form and benefit from associations with arbuscular mycorrhizal fungi (AMF; Burke et al., 2018; Whigham, 2004). Soil chemistry properties including pH and nitrogen heterogeneity are likely to influence mycorrhizal communities themselves (Coughlan, Dalpé, Lapointe, & Piché, 2000; Dumbrell, Nelson, Helgason, Dytham, & Fitter, 2010; Kluber et al., 2012), as well as the relationship between plants and mycorrhizae (Johnson, Graham, & Smith, 1997). More research is needed to assess whether plant–microbe interactions could scale up to contribute to the positive HDRs we observed in summer.

### 4.3 Negative heterogeneity–diversity relationships: potassium and phosphorus heterogeneity in spring

In spring, we found that plot‐level richness decreased with increasing potassium and phosphorus heterogeneity: both negative HDRs (Figure 4e,f). Negative HDRs can arise due to dispersal limitation (Lundholm, 2009). If spring species that differentiate across potassium and phosphorus conditions are also dispersal limited, an inability to arrive and fill available niches could lead to low richness in plots with high abiotic heterogeneity. Alternatively, if more heterogeneous plots contain subplots that can support fewer species, this too would explain the appearance of negative HDRs (Yang et al., 2015). Indeed, mean phosphorus in spring is positively correlated with phosphorus heterogeneity (Figure 3). This means heterogeneous plots tend to include higher phosphorus concentrations, which could conceivably speed competitive exclusion (Tilman, 1982) and lead to lower plot‐level richness (e.g., in Figure 4c,d,j).

### 4.4 The available energy hypothesis: calcium, nitrogen, and other measures of habitat quality

When species respond to the magnitude rather than the heterogeneity of an abiotic factor, increasing the magnitude or “available energy” (AE) of that factor may either increase or decrease species richness. Theory supports both of these possibilities: increasing the availability of a limiting nutrient can lead to faster competitive exclusion (Clark & Tilman, 2008; Tilman, 1982), as mentioned above. Alternately, increasing the level of a factor that is favorable to many different species, can increase the diversity of species that can thrive at that location (Lundholm, 2009). Our results cannot speak to mechanism, but we observed both patterns. Richness decreased with increased nitrogen availability in beech and Oak forests in spring (Figure 4j), and across plots in summer with increasing potassium and phosphorus availability (Figure 4c,d). Conversely, in spring, plot‐level richness increased across mixed forests with increasing nitrogen (Figure 4j). Forb richness also increased with increasing calcium availability in both seasons and at both spatial scales (Figures 4a,b and 5a,b). The strength and persistence of this particular trend throughout our study, as well as its prevalence in the literature (Bellemare et al., 2005; Burton et al., 2011; McEwan & Muller, 2011; Peet et al., 2014), suggests it is worth further consideration. We note that mean calcium is also highly correlated with pH and potassium variability (Figure 3).

### 4.5 Effects of scale

To determine whether the response to resource availability depended on spatial scale, we also identified the best statistical models for subplot‐level species richness using sampled subplot conditions (*α_j_*) as abiotic predictors and asked how results changed compared to plot‐level relationships. In both seasons, subplot‐level richness and plot identity were strongly correlated (step B conditional‐*r* ^2^ = 90% in spring and summer). Plot‐ and subplot‐level richness were also strongly positively correlated (*r* ^2^ = 84% and 88%). In other words, species‐rich plots had species rich subplots. Since species richness was so strongly correlated at the plot‐ and subplot‐levels, and since calcium availability was an important predictor of plot‐level richness, we thought that calcium availability would also be related to forb richness at the subplot level.

Interestingly, we found that changes in mean calcium within plots did explain additional variation in species richness (compared to models without abiotic predictors in step B; ∆AIC_C_ = 12.0 in spring and 24.7 in summer); however, this relationship was much stronger in summer (slope = 0.73) compared to spring (slope = 0.37; Figure 5a,b). In spring, subplot‐level richness did increase with increasing calcium (Figure 5a), but the relationship was weak, and most of the subplot‐level variance was only explained by accounting for plot identity (marginal‐*r* ^2^ = 11%, conditional‐*r* ^2^ = 88%; Table 4). Thus, in spring, spatial scale mattered: calcium availability explained patterns of plot‐level richness better than patterns of subplot‐level richness. In summer, both plot‐ and subplot‐level richness increased with increasing mean calcium (Figures 4b and 5b). What could cause seasonal differences in scale‐dependent effects of calcium is an interesting question for future studies.

### 4.6 Variance partitioning and the importance of forest community type

In both seasons, the variance in species richness across plots was attributed largely to mean abiotic conditions, regardless of forest type (Figure 6a). The variance in species richness explained within each forest, however, was attributed to the heterogeneity in abiotic conditions of different factors (Figure 6b,c and Table 5).

In spring, most of the variability in species richness was attributed to potassium heterogeneity in both Beech–Maple and Floodplain forests (Figure 6b and Table 5). In summer, most of the variability in species richness was attributed to pH heterogeneity in Beech–Maple and Oak forests, and to heterogeneity in cation exchange capacity in Mixed forests (Figure 6c and Table 5). Thus, we find evidence that plot‐level abiotic heterogeneity can drive patterns of species richness (Figure 6b,c), but that the identity of the important abiotic factor may change with season and forest community type (Table 5).

### 4.7 Limitations and future directions

Patterns of richness and composition within forest type could be confounded by differences in size, location (e.g., more urban or rural), and/or invasive species and deer population density (Eysenbach & Hausman, 2013) across land holdings. To control these confounding variables and test proposed mechanisms, it is necessary to confirm the drivers of increased species richness experimentally.

We also cannot discern from our data whether species are stably coexisting (due, e.g., to niche differentiation) or whether they are simply co‐occurring (due to mass effects, high available energy, slow competitive exclusion, etc.). Pending further empirical investigation, we think it is useful to consider coexistence and species richness, although clearly related, as potentially arising via separate processes.

There was a great deal of correlation between abiotic factors in both spring and summer (Figure 3), which we addressed by allowing only uncorrelated (<40%) combinations of abiotic factors into a model, thus excluding information about potentially confounding correlations. As such, there were many nearly equivalent models during the model selection process. Qualitatively, our results provide a framework for understanding the joint contributions of abiotic conditions across plots and abiotic heterogeneity within forests to patterns of forb species richness (Figure 6), but larger sample sizes within forest type are needed, as are experiments that disentangle correlated variables (e.g., between calcium availability and pH, calcium availability and potassium heterogeneity; Figure 3). To clarify how species richness is structured by different abiotic factors, we also hope that the relationships found at different study levels will be able to guide future experiments designed to test the hypotheses presented above.

## 5 CONCLUSIONS

The abiotic component of what shapes herbaceous layer forb communities is complicated, but the large amount of variation explained across study levels justifies further study and discussion of this complexity. We underscore the importance of considering mean abiotic conditions and heterogeneity simultaneously across multiple abiotic factors. In particular, we show that patterns of species richness vary across forest types according to abiotic quality, as well as within forest type according to abiotic heterogeneity. Connecting patterns associated with the abiotic mean and heterogeneity back to theoretical insights allowed us to suggest which mechanisms could be structuring plant communities at different spatial scales and seasons. Future empirical work in herbaceous forb communities should focus on using experiments to pin down the mechanism underlying plot‐level increases in richness with increasing nutrient availability, and investigating potential interactions between biotic processes and abiotic drivers within plots; for example, whether abiotically mediated microbial communities could be driving fine‐scale patterns of forb richness in different ways in different forests.

## CONFLICT OF INTERESTS

The authors report no conflict of interests.

## AUTHOR CONTRIBUTIONS

S. Catella conducted the spring survey and S. Eysenbach conducted the summer survey. All statistical analyses were drafted and conducted by S. Catella. All authors contributed to the drafting and revising of the final manuscript.

## Data Availability

Data available from the Dryad Digital Repository: https://doi.org/10.5061/dryad.kp3cb17
